# Evaluation of hydraulic fracturing using machine learning

**DOI:** 10.1038/s41598-025-12392-x

**Published:** 2025-07-24

**Authors:** Ali Akbari, Ali Karami, Yousef Kazemzadeh, Ali Ranjbar

**Affiliations:** Department of Petroleum Engineering, Faculty of Petroleum, Gas, and Petrochemical Engineering, Persian Gulf University, Bushehr, Iran

**Keywords:** Hydraulic fracturing, Machine learning, Random forest, Support vector machine, Neural networks, PKN model, fracture propagation, hydrocarbon production, Chemical engineering, Energy science and technology, Engineering

## Abstract

Hydraulic fracturing (HF) is a pivotal technique in the oil and gas industry, aimed at enhancing hydrocarbon recovery by increasing reservoir permeability through high-pressure fluid injection. Despite its effectiveness, traditional methods used to evaluate HF performance often struggle to capture the complex, nonlinear interactions among operational and geological parameters. This study presents a comprehensive machine learning (ML)-based framework to address this challenge by predicting HF efficiency using three widely used algorithms: Random Forest (RF), Support Vector Machine (SVM), and Neural Networks (NN). The novelty of this research lies in the combined application of advanced statistical characterization and comparative ML modeling over a large-scale dataset comprising 16,000 records. Key statistical metrics, including mean, median, variance, skewness, and quartiles, were used to explore data distribution and inform model training. Additionally, the study uniquely evaluates model robustness across varying train/test data ratios (from 0.1 to 0.9), providing deeper insights into algorithm performance stability. Among the tested models, RF outperformed others by achieving the highest coefficient of determination (R^2^ = 0.9804), alongside the lowest Mean Absolute Deviation (MAD) and Root Mean Square Error (RMSE) for both training and testing phases. These results demonstrate RF’s capability in handling complex subsurface data with high accuracy and low computational cost. The proposed framework not only enhances predictive accuracy in HF evaluation but also serves as a practical tool for optimizing fracturing design and decision-making in field operations. This integrated approach represents a step forward in applying artificial intelligence for data-driven reservoir engineering and contributes to the advancement of intelligent hydraulic fracturing practices in heterogeneous and data-rich environments.

## Introduction

Modern hydraulic fracturing operations involve not only the initiation and propagation of fractures but also the careful design of treatment schedules, fluid composition, and proppant transport dynamics^1,2^. The selection of fracturing fluid—ranging from slickwater to gel-based systems—is tailored to reservoir characteristics such as permeability, mineralogy, and clay content^3,4^. Furthermore, the use of fiber optics, microseismic monitoring, and real-time pressure diagnostics allows engineers to visualize fracture propagation and adjust treatment in real-time^5,6^. These innovations have significantly improved the control and efficiency of fracturing jobs. However, predicting the final fracture geometry, stimulated reservoir volume, and production response remains a major challenge due to subsurface uncertainties and complex rock-fluid interactions^7^. This complexity has encouraged the integration of data-driven approaches like machine learning to complement conventional physics-based models and enhance the design and evaluation of HF treatments^8^.

Hydraulic fracturing (HF) has emerged as one of the most transformative technologies in the development of unconventional hydrocarbon resources, such as shale oil and gas. By injecting high-pressure fluids into low-permeability formations, HF induces fractures in the rock matrix, thereby significantly enhancing permeability and hydrocarbon flow. Over the past two decades, this technology has played a central role in increasing global oil and gas production, particularly in regions like North America, where tight reservoirs dominate energy portfolios^9–15^.

Despite its widespread application, HF remains a complex and cost-intensive process. The efficiency of HF operations is influenced by a multitude of interrelated factors, including reservoir heterogeneity, fluid properties, proppant characteristics, injection parameters, and geomechanical conditions^16–24^. Traditional physics-based simulation tools, while useful, often fail to capture the nonlinear and high-dimensional nature of these interactions, leading to suboptimal designs and predictions. As a result, there is a growing interest in data-driven approaches that can effectively model the complexity of HF systems and guide decision-making processes^24–28^.

Recent advancements in data acquisition technologies have generated a large volume of operational and geological data during HF operations. These datasets provide unprecedented opportunities for analyzing and predicting fracturing outcomes. However, traditional analytical methods often face limitations when addressing the complexity and nonlinearity inherent in such data. This highlights the need for innovative solutions capable of processing, analyzing, and extracting actionable insights from them.

Machine learning (ML), a subset of artificial intelligence, has emerged as a powerful tool for addressing complex challenges across various domains, including energy systems^29–34^. In the context of HF, ML algorithms can process large datasets, identify patterns, predict fracturing efficiency, and optimize operational parameters^6,35–37^. While previous studies have explored the application of ML within this domain, a significant gap remains in understanding the performance of different models under varying geological and operational conditions.

In 2024, Khouly et al.^8^ conducted a study and concluded that fracture geometry and conductivity are fundamental factors in optimizing HF operations, especially in zones adjacent to water-bearing or gas-bearing formations. This study utilized an Artificial Neural Network (ANN) model and data from 59 HF treatments in the Western Desert of Egypt. The data were divided into 70% for training, 15% for validation, and 15% for testing. After multiple trials, the optimal ANN architecture, such as the number of hidden layers and neurons, was determined. The results of this research demonstrated that the ANN model, with a correlation coefficient of 0.93, achieved performance comparable to conventional fracture simulation software.

In 2024, Khan et al.^38^ conducted a study in which a physics-based dataset with 62 parameters was developed and transformed into a ML model. The results of this study showed that the transfer learning method successfully improved predictive performance across all outputs, with an average improvement of 15.12% in root mean square error (RMSE) and 15.88% in mean absolute percentage error (MAPE) when compared to training on real data. Additionally, particle swarm optimization (PSO) contributed to increased production, with optimized values 14.2% higher than the initial predictions. In 88% of the cases, the optimized values aligned with the real data.

In 2023, Li et al.^39^ investigated the application of ML for production forecasting in oil and gas fields. Due to its low computational cost, ML is widely used in production prediction for these fields. This study focused on the productivity prediction of shale gas wells with HF in the Changning area of the Sichuan Basin. Four different methods, including Multiple Linear Regression (MLR), Support Vector Machine (SVM), Random Forest (RF), and ANN, were evaluated. The results showed that the MLR and SVM methods performed poorly with relatively high errors (> 15%), whereas the ANN and RF methods yielded better results, with RF having a median error of around 12% and ANN having the smallest error (< 10%). After the production prediction, PSO was applied to improve gas production, leading to nearly a two-fold increase in gas output. This study provides valuable insights for shale gas production through HF in the Changning area.

This study aims to fill this gap by developing and evaluating an ML-based framework for HF performance prediction using a large-scale dataset (16,000 records) and three popular algorithms: RF, SVM, and Neural Networks (NN). A comprehensive statistical analysis is conducted to characterize input data, while model performance is evaluated across various train/test data splits to ensure robustness. The results demonstrate the superior performance of the RF model in terms of accuracy and stability, offering valuable insights for optimizing HF design and improving reservoir management practices. This integrated approach not only contributes to the advancement of intelligent hydraulic fracturing but also provides a practical tool for real-world applications in heterogeneous and data-rich environments.

## Literature review

### Hydraulic fracturing

**Fig. 1:**
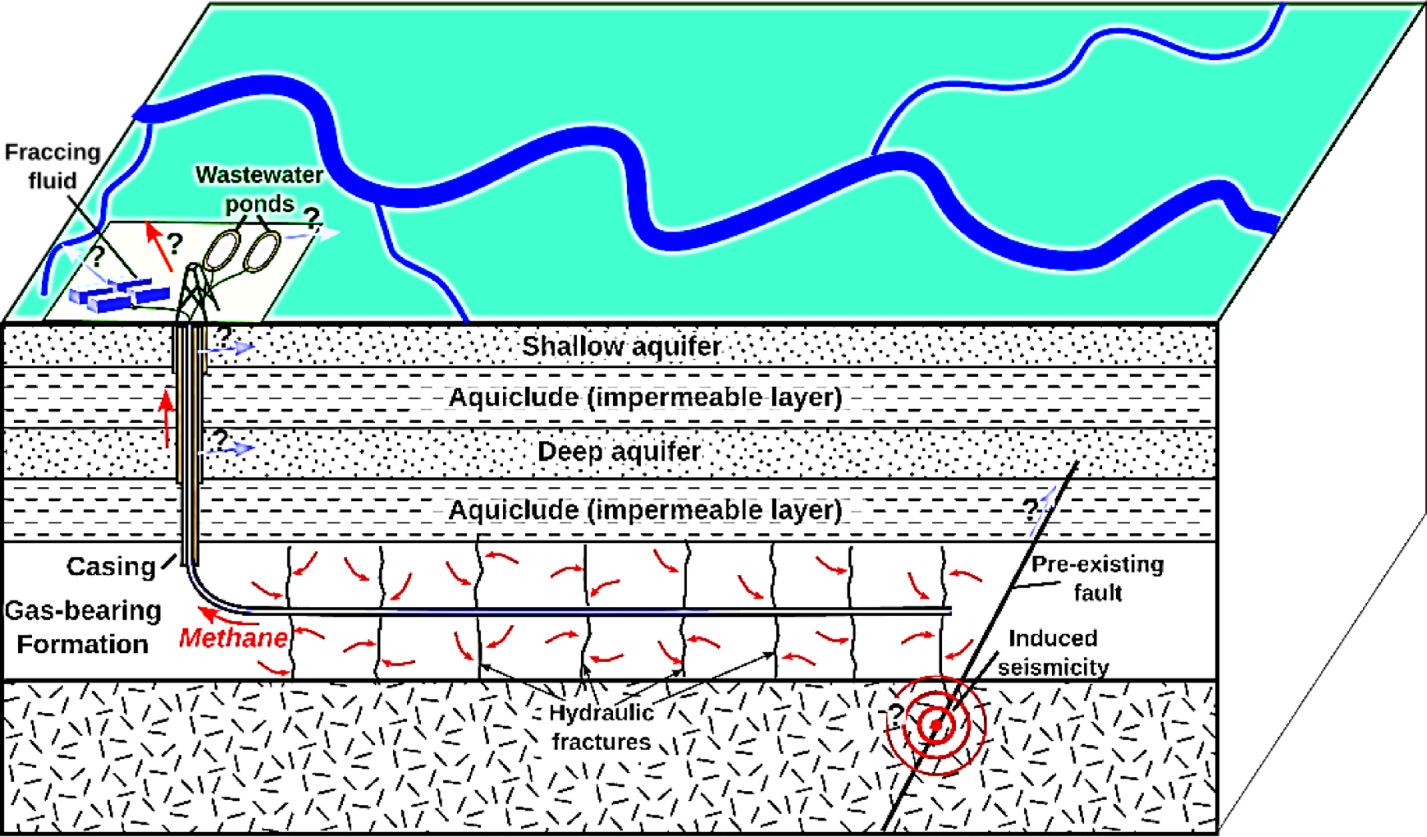
Schematic depiction of HF.

HF, commonly known as fracking, is an advanced technique used in the drilling and extraction of oil and gas resources. In this process, rock formations are fractured by injecting a high-pressure fluid into the wellbore^40–47^. This fluid typically consists of water, sand, and other proppant materials, which are used to create fractures and cracks in the rock structure^48–51^. The goal of HF is to create pathways within the rock layers that facilitate the easier and faster flow of natural gas, crude oil, and brine from the reservoir to the surface (Fig. 1).

A key aspect of the HF process is that, once hydraulic pressure is applied to the reservoir and fractures are formed, the injected fluid is removed from the wellbore^52–54^. However, fine sand particles or similar materials, known as proppants, remain within the fractures^55–58^. These proppants effectively help keep the fractures open and prevent them from closing, ensuring continued hydrocarbon flow to the surface and improving the efficiency of extraction operations^59,60^.

HF plays a critical role in the exploitation of unconventional oil and gas resources, particularly in shale oil and gas fields, which are difficult to extract using conventional methods^61–63^. This technique has made vast reserves of shale gas and oil in various regions of the world, including the United States, a crucial source of energy^64–66^. Consequently, HF not only contributes to increasing oil and gas production but is also recognized as a key factor in ensuring global energy security.

This process consists of four main stages, each playing a crucial role in fracturing the rock structure and creating optimized pathways for hydrocarbon flow:

### Perforation

In this stage, controlled explosions are conducted within the well using a specialized tool known as a Perforating Gun. The purpose of these explosions is to create small fractures in the wellbore, which serve as initial pathways for the flow of fluids into the reservoir. Typically, perforation occurs in specific sections of the well that are suitable for HF fluid injection. After this operation, hydraulic pressure is applied to the reservoir, causing these fractures to open more significantly, allowing fluids and proppants to penetrate deeper into the rock formation.

### Injection of fluid and initial fracture creation

During this stage, HF fluid is injected into the wellbore. The fluid is injected under high pressure, which initiates the creation of primary fractures in the rock. Initially, these fractures are small enough to allow fluid movement within the reservoir, but as the operation progresses, the fractures expand, providing greater access to the hydrocarbon-bearing rock. The hydraulic fluid typically consists of water, chemicals, and sand, which help generate high pressure and stimulate the fracturing of the rock formation.

### Continued fluid injection and proppant injection

Following the creation of the initial fractures, continued injection of hydraulic fluid is necessary to further open the fractures and access a larger portion of the reservoir. During this stage, proppants (such as sand or aluminum oxide) are injected along with the fracturing fluid. These proppants are crucial for maintaining the open fractures and preventing them from closing after the hydraulic pressure is reduced. The proppants effectively keep the fractures open, facilitating the flow of hydrocarbons from the reservoir into the wellbore.

### Ceasing fluid injection and flowback of fracturing fluid

In this final stage, the injection of fluid is halted, and the fracturing fluid that was pumped into the wellbore during the operation begins to flow back to the surface. This fluid, typically containing water, chemicals, and spent proppants, is returned to the surface and removed from the well. The flowback of the fracturing fluid indicates that the fractures have been successfully opened and the proppants are in place, allowing for the natural flow of hydrocarbons from the reservoir into the wellbore. After this stage, the well is now ready for hydrocarbon production, which can occur through natural flow or enhanced recovery methods such as pumping or gas pressure assistance.

The PKN model (Perkins-Kern-Nordgren) is a fundamental model for describing hydraulic fracturing in oil and gas formations. This model is specifically developed to describe long and narrow fractures in reservoirs with limited thickness and is based on simplified assumptions about fluid flow and fracture geometry. In this model, the fracture propagates either horizontally or vertically within a homogeneous formation, with the fracture width (opening), length, and height being the primary parameters under investigation.

The PKN (Perkins-Kern-Nordgren) model for hydraulic fracturing in the oil industry is described as follows. Using the provided parameters, the equation takes the following form:

1 $$\:\sigma\:=C: \epsilon$$

2 $$\:\nabla\:\sigma\:+\rho\:g=\rho\:\ddot{u}$$

The Cauchy stress tensor (σ), linear strain tensor (ε), and elastic tensor (C) are determined by the Poisson’s ratio (ν). Additionally, local rock density (ρ), gravity acceleration (g), and displacement (u) are also considered as significant factors influencing rock deformation.

3 $$\:w=\frac{\left(1-v\right)}{G}\sqrt{\left({h}^{2}-4{z}^{2}\right)}(p-{\sigma\:}_{0})$$

h represents the fracture height, z denotes the vertical coordinate, p stands for fluid pressure, and σ0 denotes the ambient stress.

4 $$\:q=-\frac{{w}^{3}}{12\mu\:}\frac{dp}{ds}$$

For fluid flow in a two-dimensional hydraulic fracture, Poiseuille’s law is expressed as follows: Here, q represents the flow rate, w denotes the fracture width, µ is the viscosity of the fracturing fluid, p stands for fluid pressure, and s is the local coordinate aligned with the tangential direction to the fracture path.

5 $$\:\frac{\partial\:p}{\partial\:x}=-\frac{64\mu\:q}{\pi\:{w}_{max}^{3}h}$$

The pressure gradient in the direction of fracture propagation is calculated for laminar flow in an elliptical tube according to the classic solution for laminar flow. Here, wmax represents the fracture width at the center.

6 $$\:\frac{\partial\:A}{\partial\:t}+\frac{\partial\:q}{\partial\:x}+{q}_{L}=0$$

The continuity equation for fluid flow, where A represents the cross-sectional area of the fracture.

**Fig. 2:**
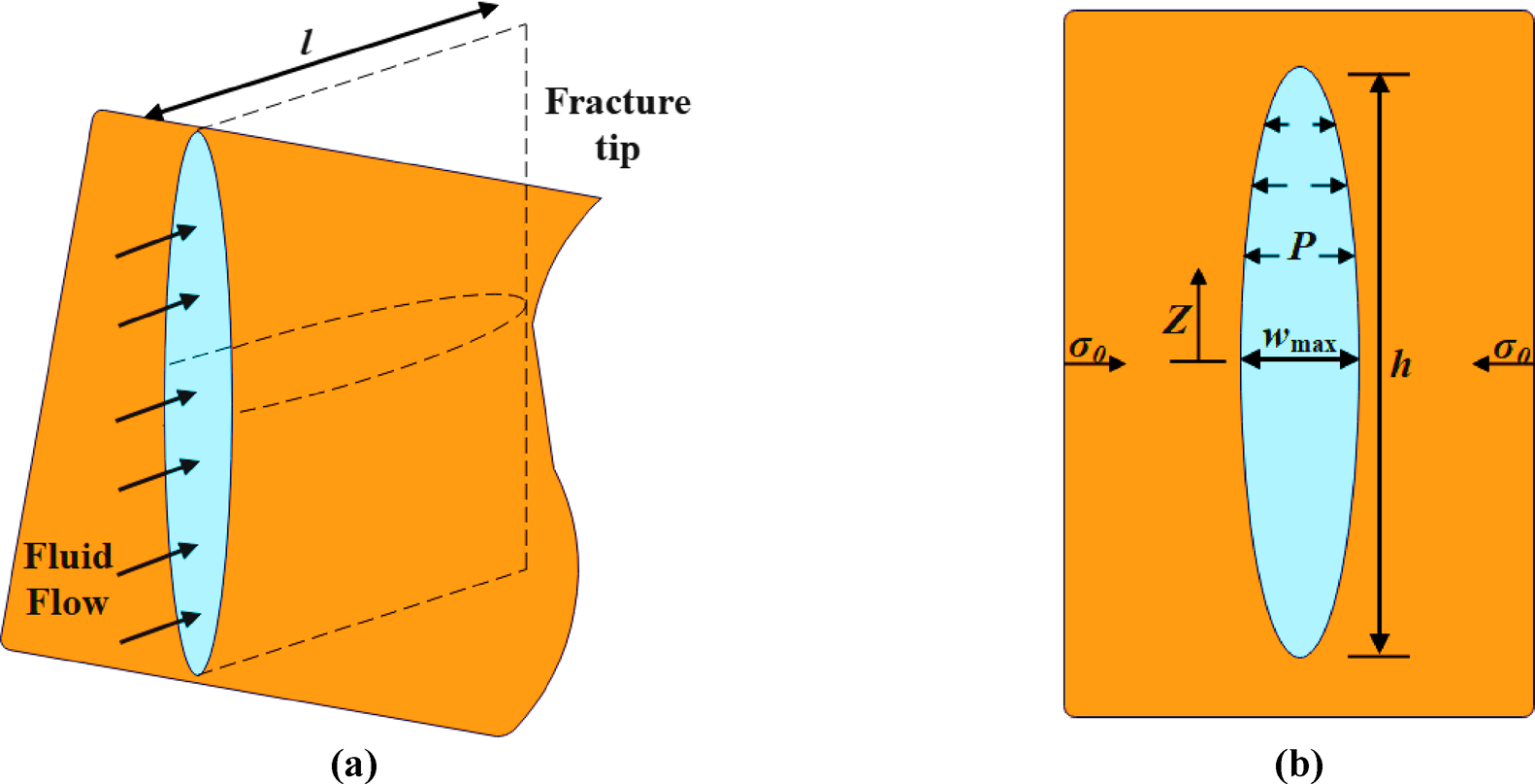
The depiction of the PKN model: (**a**) illustrating the model setup, and (**b**) showcasing the assumption of plane strain on the vertical section.

For a clearer understanding of hydraulic fracturing performance, refer to Fig. 2. Figure 2a presents a schematic side view of the geological layers within a hydrocarbon reservoir. This view illustrates the stratigraphy of the subsurface, the drilled well path, and the vertical extent of the fractures, allowing for a better perception of fracture depth and their propagation across different formations. It provides valuable insights into how fractures interact with the surrounding rock layers at various depths.

In contrast, Fig. 2b offers a closer and more detailed frontal view of the formation where the hydraulic fracturing process has taken place. This perspective highlights the specific zone of fracturing and focuses on the mechanics of fracture propagation. It illustrates the high-pressure injection of fracturing fluid and the placement of proppants (fracture-holding particles) within the created fractures. These details help engineers and researchers analyze fracture development, stability, and their impact on enhancing reservoir permeability.

Together, these two illustrations complement each other by offering dual perspectives—one from the side and one from the front—providing a complete visual representation of the hydraulic fracturing process and its effects on the subsurface formation. They serve as essential tools for simulating, understanding, and optimizing fracture design in real-world reservoir operations.

**Table 1:** Includes a collection of articles on the application of ML methods in optimizing and predicting the performance of HF operations. It examines the methods and results obtained from each of these articles.

Row	Author(s)	Year of Publication	Title	ML Technique	Best Final Result	Results of the Article
1	Khouly et al.^8^	2024	Integration Between Different Hydraulic Fracturing Techniques and Machine Learning in Optimizing and Evaluating Hydraulic Fracturing Treatment	ANN	Correlation coefficient (R) of 0.93	The ANN model showed promising results for predicting fracture geometry, with a high correlation between predicted and actual fracture geometry parameters. The model outperformed common fracture simulation software.
2	Khan et al.^38^	2024	Physics-Informed Machine Learning for Hydraulic Fracturing—Part II: The Transfer Learning Experiment	Neural Networks with Transfer Learning	Average RMSE improvement of 15.12% and MAPE improvement of 15.88% compared to real-data-trained approach	Transfer learning demonstrated enhanced predictive capabilities across five outputs: fluid efficiency, pad ratio, proppant mass, maximum proppant concentration, and dimensionless productivity index (JD). The optimized values improved production, with 88% of instances showing optimal results.
3	Wang et al.^67^	2024	Characterization of natural fracture development in coal reservoirs using logging machine learning inversion, well test data and simulated geostress analyses	SVM, RF, XGBoost, BPNN	BPNN: 95.8% accuracy	BPNN outperformed other machine learning models (SVM, RF, XGBoost) in predicting fracture development, with an accuracy of 95.8%. The model successfully integrated geostress data with fracture prediction, showing good agreement with actual field data.
4	Luo et al.^68^	2024	Production Forecast for Multistage Hydraulically Fractured Shale Gas Well Based on Integration of Domain Knowledge and Deep Learning Algorithm	Deep Learning (GRU model with Mask layer)	MRE of 11.7%, 77.4% lower than traditional models	The deep learning model integrated domain knowledge and outperformed traditional models, achieving a much lower mean relative error (MRE). The Mask layer improved data efficiency, enhancing the accuracy of the production forecast.
5	Li et al.^39^	2023	Prediction of Shale Gas Production by Hydraulic Fracturing in Changning Area Using Machine Learning Algorithms	MLR, SVM, RF, ANN	RF: ~12% median error, ANN: <10% error	The RF and ANN models were the most accurate for predicting shale gas production, with RF having a median error of ~12% and ANN having the smallest error (<10%). Particle swarm optimization improved production by approximately two times.
6	Yue et al.^69^	2022	Prediction of effective stimulated reservoir volume after hydraulic fracturing utilizing deep learning	Branched Deep Neural Network (B-DNN)	97% agreement with actual field data	The B-DNN model outperformed CNN and RNN models in predicting the effective stimulated reservoir volume (SRV) after hydraulic fracturing, with high accuracy and efficiency. The model was less time-consuming compared to CNN and RNN.

## Methodology

### Peculiarities of the applied machine learning methods

The machine learning framework developed in this study exhibits several distinctive features that set it apart from conventional approaches applied in HF analysis. First and foremost, a large-scale dataset comprising 16,000 data records was utilized, which is significantly larger than the datasets used in many previous studies. This extensive dataset enhances the robustness and generalizability of the models, allowing them to better capture the underlying patterns and interactions among HF parameters.

Secondly, the study integrates comprehensive statistical analysis—including metrics such as mean, variance, skewness, kurtosis, quartiles, and data visualization via box plots and violin plots—to better understand the distribution and variability of the input variables. Such detailed preprocessing is often overlooked in many data-driven HF studies, yet it plays a crucial role in improving model accuracy and interpretation.

Another unique aspect of the proposed methodology is the evaluation of model performance across multiple train/test ratios, ranging from 0.1 to 0.9. This systematic approach provides a deeper understanding of how data availability affects model performance and stability. The analysis of R^2^ values across these splits, supported by multiple independent runs for each model, offers insights into the consistency and reliability of different algorithms under varying data constraints.

Furthermore, the models were developed using domain-specific parameters such as fracture height, fracture length, fluid viscosity, and injection time, which are directly derived from the governing physical equations of HF. This integration of physics-based variables into ML models enhances their relevance to real-world operations and bridges the gap between data-driven techniques and conventional engineering understanding.

Finally, by comparing three well-established algorithms—RF, NN, and SVM—under identical conditions, the study provides a fair and comprehensive evaluation of model capabilities, with RF demonstrating superior performance in terms of accuracy and error minimization.

### Data preprocessing

In this study, the data were initially plotted using analytical formulas, and the assumptions considered for modeling were outlined. The data were then analyzed to examine patterns and key features. Subsequently, using the MATLAB programming language libraries — a common tool in engineering and computer science — the SVM, NN, and RF methods were implemented, and the datasets were analyzed, organized, and sorted using Microsoft Excel. These algorithms are among the most widely used ML techniques for prediction and data analysis. Using these algorithms, the R^2^ value was calculated, which serves as a metric for evaluating the accuracy of prediction models. The dataset comprised 16,000 data points with 4 input variables. Table 2 presents the statistical insights related to the dataset and its distribution.

The required statistical information is also presented in Table 2.

**Table 2:** Data statistics.

	$\:\varvec{\mu\:}$	$\:\varvec{h}$	$\:\varvec{X}$	$\:\varvec{t}$
Max	999.798	983.890	1997.659	179.998
Min	50.437	262.659	1.305	30.020
Range	949.361	721.231	1996.354	149.978
Median	532.754	631.755	982.796	105.561
Q1	287.926	445.490	500.247	65.847
Q3	772.414	811.492	1492.191	143.102
Mean	528.955	629.039	987.441	104.876
Variance	76222.545	43514.210	330749.001	1935.455
Skewness	− 0.020	− 0.033	0.002	− 0.009
Kurtosis	− 1.200	− 1.208	− 1.184	− 1.234

In this paper, several key parameters for analyzing fractures in the HF process are examined. These parameters are each represented by a specific symbol: $\:\mu\:$ (mu) denotes the viscosity of the fracturing fluid (in centipoise), $\:h$ represents the crack height, $\:t$ indicates the injection time, and $\:X$ corresponds to the crack length. Understanding and accurately measuring these variables are crucial for interpreting the study’s results, as well as for developing predictive models and strategies. This is particularly important in HF, where the interaction between fluid flow and fracture propagation has a significant impact on the process outcomes.

**Fig. 3:**
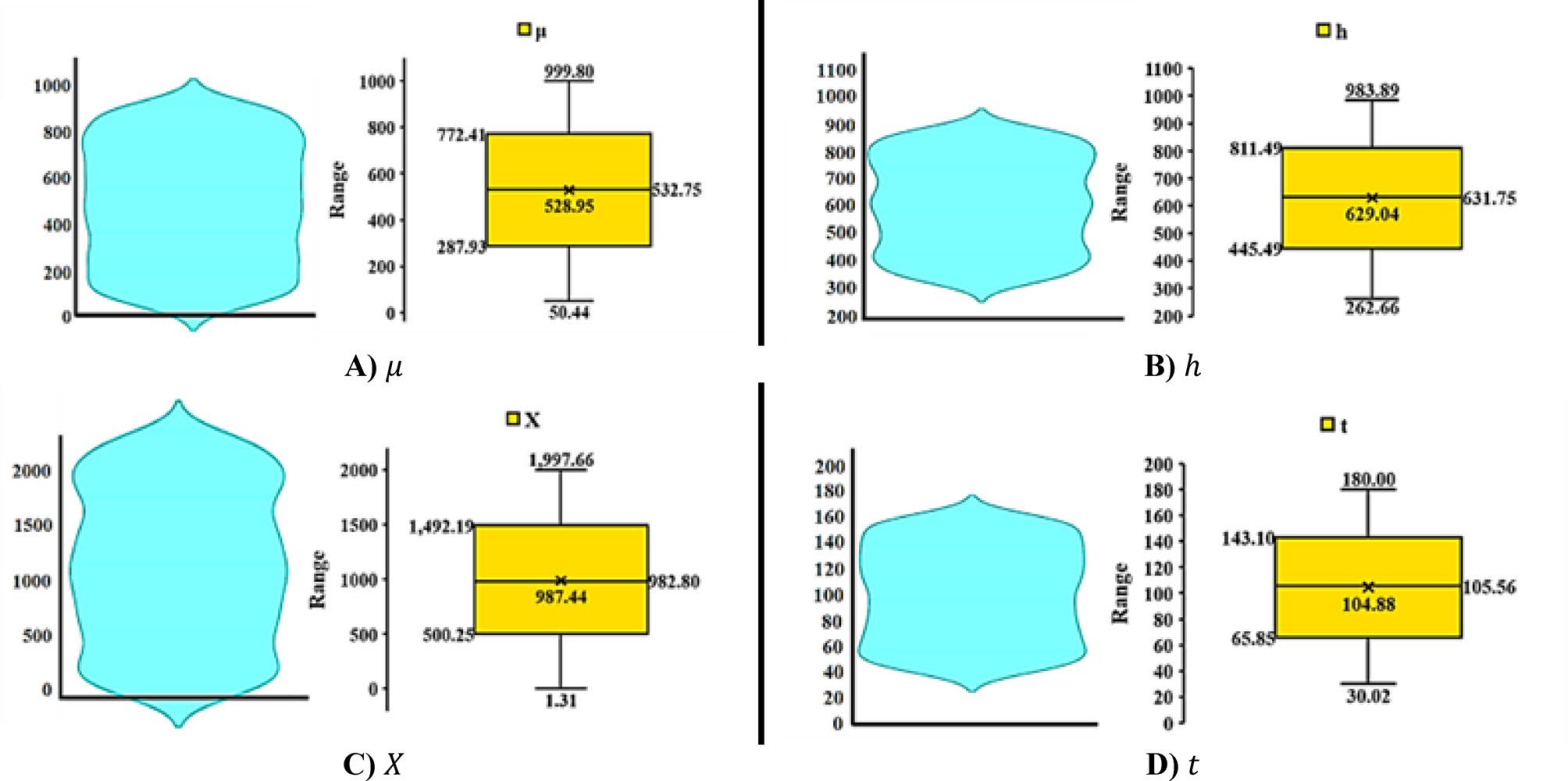
Box-plot and Violin Plots of HF parameters.

The data obtained were calculated using Eqs. 1–6. Subsequently, machine learning methods were applied to analyze the data with the corresponding inputs. The data range is also presented in Table 2; Fig. 3.

The provided table presents a statistical analysis of the HF process, aimed at evaluating its performance. This analysis includes various statistical parameters such as maximum, minimum, range, median, first quartile (Q1), third quartile (Q3), mean, variance, and skewness.

The median, as a significant statistical measure, divides the dataset into two equal parts, with half of the data points are below the median and the other half are above it. This measure is particularly valuable in analyzing datasets containing outliers, as it is minimally affected by extreme values. To calculate the median, the data must first be arranged in ascending order. If the number of data points is odd, the median corresponds to the middle value. Conversely, if the number of data points is even, the median is calculated as the average of the two middle values.

7 $$\:Median\:\left\{\begin{array}{c}{x}_{\left(\frac{n+1}{2}\right)}\:For\:an\:odd\:EquationNumber\:of\:data\:points\:n\:\\\:\frac{{x}_{\left(\frac{n}{2}\right)}+{x}_{(\frac{n}{2}+1)}}{2}\:\:\:For\:an\:even\:EquationNumber\:of\:data\:points\:n\end{array}\right.$$

Quartiles are statistical measures that divide a dataset into four equal parts. The Q1 marks the value below which 25% of the data points are located, indicating that the remaining 75% lie above it. Conversely, the Q3 identifies the value below which 75% of the data points fall, with 25% positioned above it. Quartiles play a crucial role in Box Plots, as they help visualize the distribution and concentration of data, offering valuable insights into its range and potential outliers.

The mean, a measure of central tendency, represents the average of a dataset. It is determined by adding all the data points together and dividing the sum by the total number of observations. However, the mean is highly sensitive to outliers, which can distort its accuracy. As a result, in datasets with high variability or extreme values, the median or quartiles may serve as more reliable indicators of central tendency.

8 $$\:Mean=\frac{1}{n}\sum\:_{i=1}^{n}{X}_{i}$$

Variance measures the degree to which data points deviate from the mean. A high variance reflects a wide distribution, indicating that the data points are spread out significantly from the mean. On the other hand, a low variance indicates that the data points are closely concentrated around the mean. Variance is a valuable tool in statistical analysis, as it quantifies the level of variability within a dataset and offers a deeper understanding of its distribution and characteristics.

9 $$\:{Population\:Variance:\:\sigma\:}^{2}=\frac{1}{n}\sum\:_{i=1}^{n}{({X}_{i}-\mu\:)}^{2}$$

10 $$\:{Sample\:Variance:\:s}^{2}=\frac{1}{n-1}\sum\:_{i=1}^{n}{({X}_{i}-\stackrel{-}{X})}^{2}$$

Skewness quantifies the asymmetry in a dataset’s distribution. A positive skewness indicates that the distribution extends towards higher values, with a larger concentration of lower values and a few extreme high values. In contrast, negative skewness suggests the distribution is elongated towards lower values, characterized by more high values and a few extreme low values. Analyzing skewness is crucial for understanding data distribution, as it reveals tendencies towards a particular direction, thereby influencing statistical interpretations and decision-making processes.

11 $$\:\text{S}\text{k}\text{e}\text{w}\text{n}\text{e}\text{s}\text{s}=\frac{n}{(n-1)(n-2)}\sum\:_{i=1}^{n}{\left(\frac{{X}_{i}-\stackrel{-}{X}}{s}\right)}^{3}$$

Each of these parameters, with their unique characteristics, plays a crucial role in describing and analyzing datasets, providing valuable insights into their distribution, variability, and asymmetry. The collected data have been carefully analyzed, and the parameters related to the HF process have been separately plotted from a statistical perspective. The analyses are visually presented using Box Plots and Violin Plots (Fig. 3), which illustrate a range of statistical indicators, including the median, Q1, Q3, mean, variance, skewness, as well as the maximum and minimum values.

Based on Fig. 3, it can be concluded that:

In this study, the descriptive statistics for four critical variables related to HF are analyzed: viscosity of the fracturing fluid (µ), height of the fracture (h), length of the fracture (X), and injection time (t). These statistics help in understanding the data distribution, variability, and behavior. The maximum and minimum values for these parameters indicate significant variability, with viscosity ($\:\mu\:$) showing a wide range, from 50.437 to 999.798, and fracture length (X) ranging from 1.305 to 1997.659. The range for fracture height ($\:h$) is also substantial, while injection time (t) has a more moderate spread.

The median, Q1, and Q3 values provide further insights into the data distribution. For $\:\mu\:$, the median is 532.754, with Q1 at 287.926 and Q3 at 772.414, indicating a moderate spread in viscosity values. Similar patterns are observed for $\:h$, $\:X$, and t, with Q1 and Q3 values suggesting varying degrees of spread in the data. The mean values for all parameters are close to their respective medians, indicating relatively symmetric distributions, with viscosity, fracture height, and length values being close to the average.

Variance analysis shows that viscosity ($\:\mu\:$) has the highest variance, reflecting its significant role in the variability of the HF process. The fracture height ($\:h$) and length ($\:X$) also exhibit considerable variance, indicating high variability in fracture dimensions. In contrast, injection time ($\:t$) has the lowest variance, suggesting more consistency in the injection process.

Skewness and kurtosis analyses show that viscosity and fracture height data are slightly negatively skewed, with values slightly concentrated toward the higher end. Fracture length and injection time distributions are nearly symmetric. The kurtosis values for all parameters are negative, indicating platykurtic distributions, which suggests fewer extreme outliers compared to normal distributions.

**Fig. 4:**
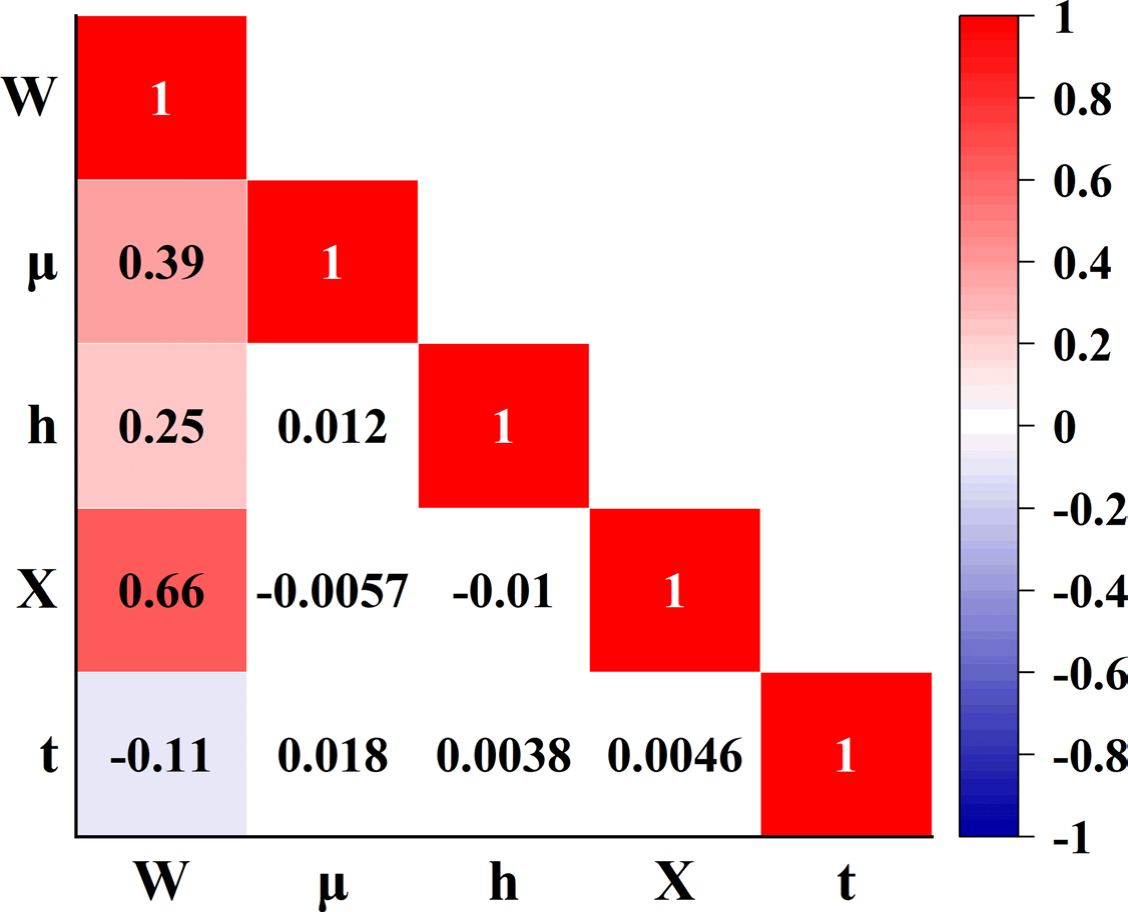
Correlation plot.

Figure 4 presents the correlation matrix among five variables—W, µ, h, X, and t—both numerically and graphically. The values within the matrix represent the Pearson correlation coefficients between each pair of variables, ranging from − 1 to + 1. Coefficients close to + 1 indicate a strong positive correlation, those near − 1 signify a strong negative correlation, and values around zero suggest no significant linear relationship. The color scheme, ranging from blue (negative correlation) to red (positive correlation), further facilitates the interpretation of these relationships. The results reveal that the strongest positive correlation occurs between variables W and X, with a coefficient of 0.66. In contrast, other variable pairs such as h and µ, h and X, as well as t with the remaining variables, exhibit very weak or negligible linear correlations. This analysis plays a crucial role in identifying influential variables and enhancing the understanding of their interrelationships.

### Machine learning methods

#### Neural network

Neural networks (NN) are one of the ML algorithms inspired by the structure and functioning of the human brain. These networks consist of interconnected nodes (neurons) that process data in layers. NNs are highly effective in solving complex problems in areas such as image recognition, natural language processing, and prediction.

A typical neural network consists of three types of layers:

- Input Layer: Receives the input data.
- Hidden Layers: Perform computations and feature extraction.
- Output Layer: Provides the prediction or final result.

**Fig. 5:**
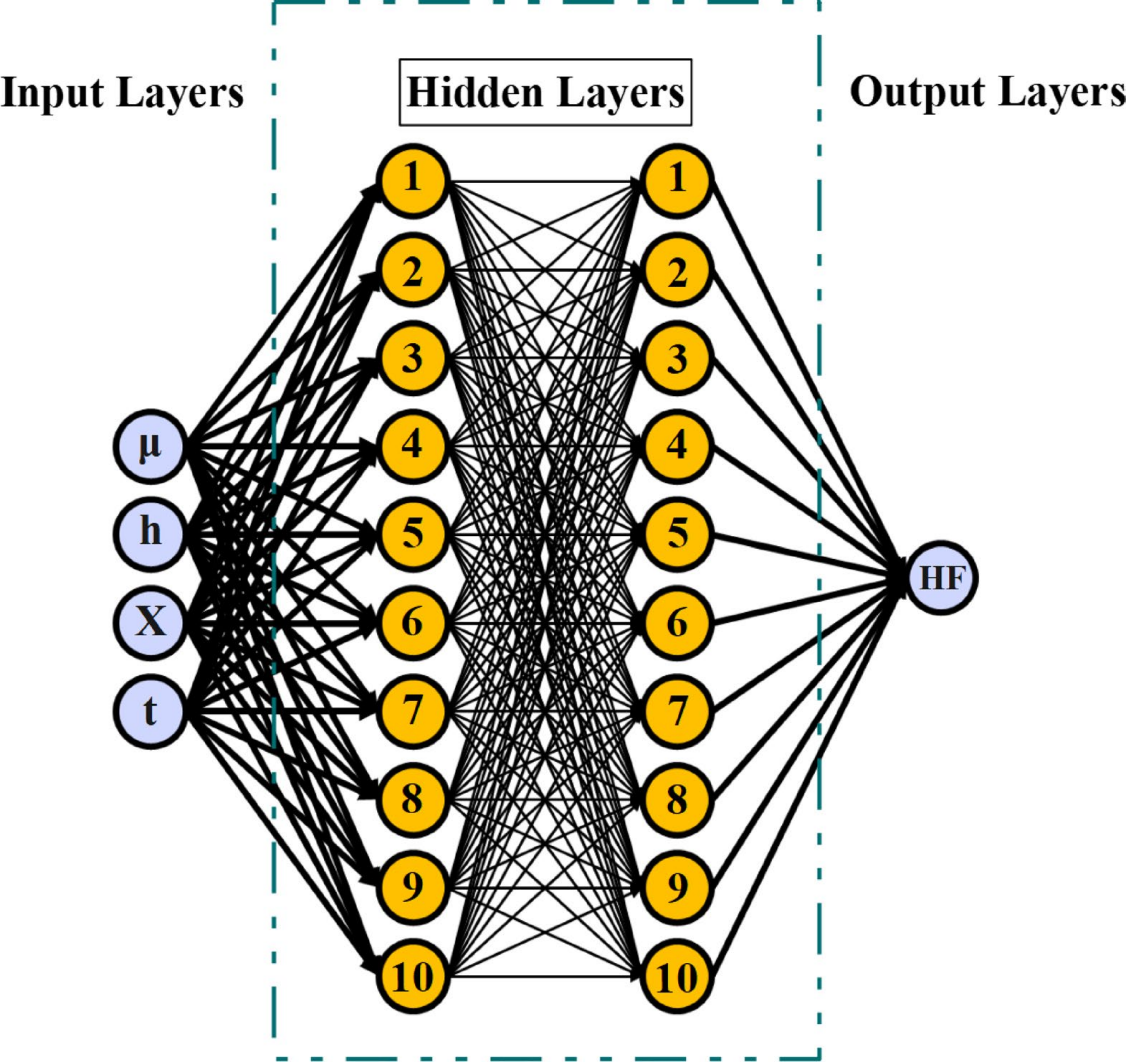
NN algorithm.

Each node in a layer is connected to nodes in the next layer via weighted connections. During training, these weights are adjusted to minimize the error between predictions and actual outputs (see Fig. 5).

Equations and Evaluation Metrics of NN.

1. Activation functions

Each neuron processes input values by applying an activation function, which determines whether the neuron should be activated. Common activation functions include:

- Sigmoid:
  12 $$\:f\left(x\right)=\frac{1}{1+{e}^{-x}}$$

Used for binary outputs, compressing values between 0 and 1.

- ReLU (Rectified Linear Unit):
  13 $$\:f\left(x\right)=\text{m}\text{a}\text{x}(0,x)$$

Commonly used in hidden layers for faster convergence.

- SoftMax:
  14 $$\:f\left({x}_{i}\right)=\frac{{e}^{{x}_{i}}}{{\sum\:}_{j=1}^{N}{e}^{{x}_{i}}}$$

Used in multi-class classification problems.

(2) Forward propagation

During forward propagation, data flows from the input layer through hidden layers to the output layer. At each neuron, the weighted sum of inputs is calculated, followed by the application of an activation function:

15 $$\:z=\sum\:_{i=1}^{n}{w}_{i}{x}_{i}+b$$

16 $$\:a=f\left(z\right)$$

Here, $\:{w}_{i}$ are the weights, $\:{x}_{i}$ are inputs, $\:b$ is the bias term, $\:z$ is the weighted sum, and $\:a$ is the activated output.

(3) Cross-Entropy loss (for classification)

17 $$\:L=-\frac{1}{n}\sum\:_{i=1}^{n}\sum\:_{j=1}^{C}{y}_{ij}\text{log}{\widehat{y}}_{ij}$$

Where $\:y$ is the true label and $\:\widehat{y}$ is the predicted probability.

(4) Backward propagation and optimization

Backward propagation adjusts the weights to minimize the loss function using optimization algorithms like Gradient Descent. The gradients of the loss function with respect to the weights are computed using the chain rule of calculus.

The weight update formula is:

18 $$\:{w}^{(t+1)}={w}^{\left(t\right)}- \eta \frac{\partial\:L}{\partial\:w}$$

Where $\eta$ is the learning rate, $\:L$ is the loss, and $\:w$ are the weights.

### Random forest

RF is a ML algorithm based on the ensemble learning technique, which combines multiple decision trees to improve model accuracy and reduce the risk of overfitting. It is applicable to both classification and regression tasks and leverages two primary techniques: Bootstrap Aggregation (Bagging) and Random Feature Selection.

In the Bagging method, multiple subsets of training data are randomly generated with replacement. Each decision tree is trained on one of these subsets, reducing the variance of the model. In the Random Feature Selection method, at each node of the tree, only a random subset of features is considered for decision-making. This approach reduces the correlation between trees and enhances the final model’s accuracy.

**Fig. 6:**
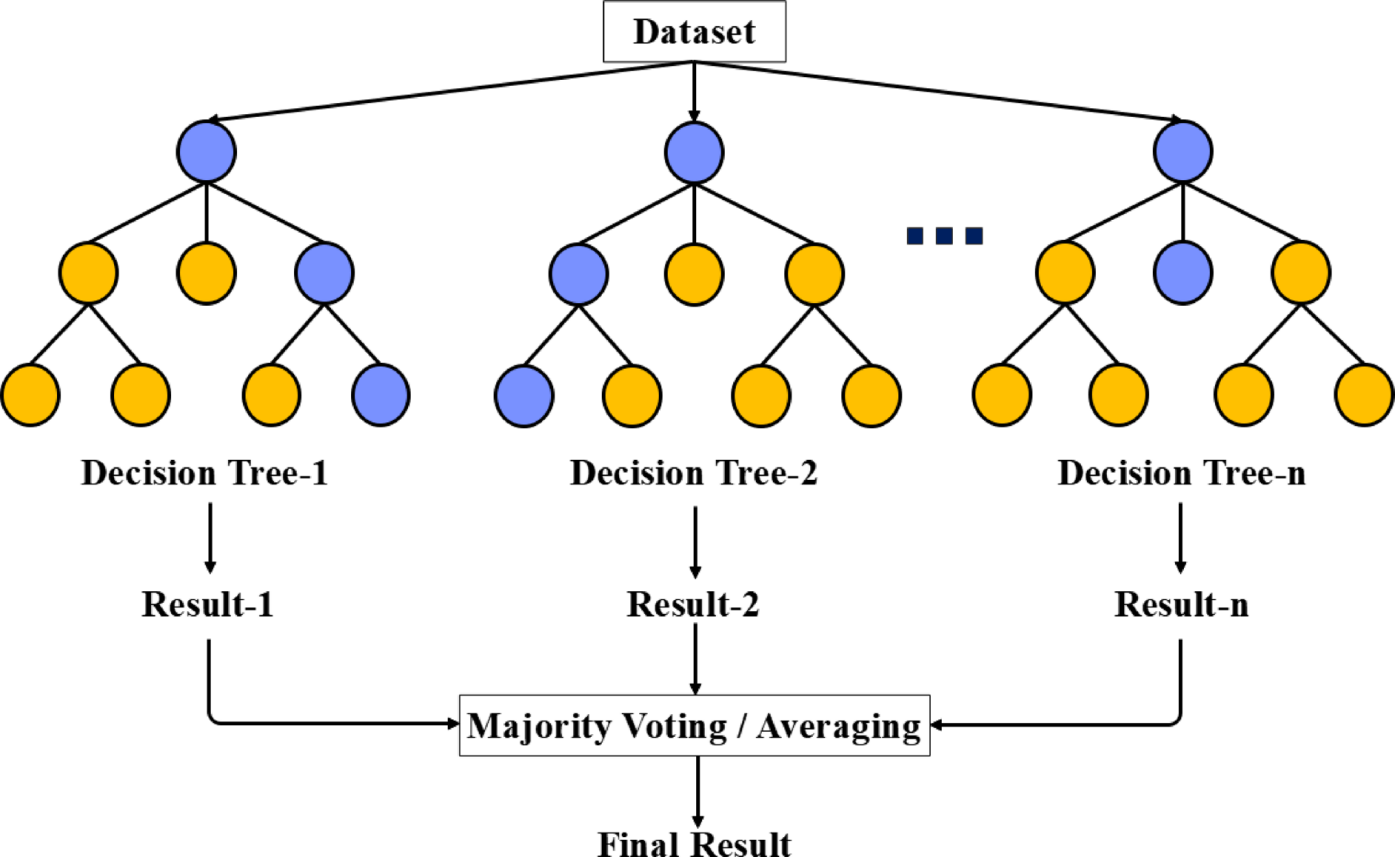
RF Algorithm.

The final prediction in RF is made using majority voting for classification tasks and by calculating the mean output of all trees for regression tasks. This combination results in a highly accurate model that is resilient to small variations in data. Furthermore, RF is highly resistant to overfitting due to its use of random data subsets and feature limitations (see Fig. 6).

Equations and Evaluation Metrics of RF.

1. Entropy (for classification)

19 $$\:H\left(S\right)=-\sum\:_{i=1}^{C}{p}_{i}{{log}}_{2}\left({p}_{i}\right)$$

Where $\:C$ is the number of classes, and $\:{p}_{i}$ is the probability of each class.

(2) Gini index (for classification)

20 $$\:G\left(S\right)=1-\sum\:_{i=1}^{C}{p}_{i}^{2}$$

(3) Prediction Aggregation:

- For classification:
  21 $$\:\widehat{y}=Mode\left\{{h}_{1}\left(x\right),\:{h}_{2}\left(x\right),\:\dots\:,\:{h}_{k}\left(x\right)\right\}$$

- For regression:
  22 $$\:\widehat{y}=\frac{1}{k}\sum\:_{i=1}^{k}{h}_{i}\left(x\right)$$

Where $\:{h}_{i}\left(x\right)$ is the prediction of the $\:i-th$ tree, and $\:k$ is the number of trees.

The implementation of the RF algorithm involves three main steps. The first step is generating random samples using Bootstrap. In this process, multiple random subsets are created from the original training dataset through sampling with replacement. These subsets serve as the training data for individual decision trees, ensuring diversity and reducing overfitting in the overall model.

The second step involves building decision trees. Each subset is used to construct a unique decision tree. At each node within the tree, a random subset of features is selected, rather than considering all features. This ensures further randomness and reduces correlation among the trees. To determine the optimal decision-making criteria at each node, metrics such as Entropy or Gini Index are used to measure the impurity or information gain.

The final step is aggregating the outputs of the trees. For classification tasks, the final prediction is based on a majority voting system, where the class predicted by the majority of trees becomes the output. For regression tasks, the final prediction is calculated by taking the mean of the outputs from all the decision trees. This aggregation method ensures a robust and accurate final prediction, leveraging the diversity of the ensemble.

#### Support vector machine

SVM is a supervised ML algorithm used for both classification and regression tasks. It aims to find the best hyperplane that separates the data while maximizing the margin between the classes. The hyperplane serves as the boundary that separates data points belonging to different classes. In two-dimensional space, the hyperplane is a line, while in three-dimensional space, it becomes a plane. The general equation of the hyperplane is as follows:

23 $$\:w\cdot\:x+b=0$$

Here, $\:w$ represents the weight vector, $\:x$ denotes the feature vector, and $\:b$ is the bias term. The margin is the distance between the hyperplane and the nearest data points from each class, known as support vectors. The objective of SVM is to maximize this margin, which enhances the model’s generalization capability.

To address non-linear problems, SVM employs kernel functions. These functions map the data into higher-dimensional spaces where it becomes linearly separable. Common kernels include the linear kernel, polynomial kernel, and radial basis function (RBF) kernel. Their equations are as follows:

- Linear Kernel:

24 $$\:K\left({x}_{i},{x}_{j}\right)={x}_{i}\cdot\:{x}_{j}$$

- Polynomial Kernel:

25 $$\:K\left({x}_{i},{x}_{j}\right)={{(x}_{i}\cdot\:{x}_{j}+C)}^{d}$$

- Radial Basis Function (RBF) Kernel:

26 $$K\left( {x_{i} ,x_{j} } \right) = {\text{exp}}\left( { - \gamma \left\| {(x_{i} - x_{j} } \right\|^{2} } \right)$$

In cases where the data is not perfectly separable, SVM uses the concept of a soft margin. This allows some data points to be misclassified. A regularization parameter, $\:C$, controls the trade-off between maximizing the margin and minimizing classification errors.

The optimization problem in SVM to find the optimal hyperplane is defined as:

27 $$min\frac{1}{2}\left\| w \right\|^{2}$$

Subject to:

28 $$\:{y}_{i}\left(w\cdot\:{x}_{i}+b\right)\ge\:1\:\:\:for\:all\:i$$

For non-separable data, slack variables ($\:{\xi\:}_{i}$) are introduced, and the optimization problem is modified as follows:

29 $$\:min\frac{1}{2}{ \left\| w \right\| }^{2}+C\sum\:_{i=1}^{n}{\xi\:}_{i}$$

This problem is typically solved using its dual form, where the objective function becomes:

30 $$\:max\sum\:_{i=1}^{n}{\alpha\:}_{i}-\frac{1}{2}\sum\:_{i=1}^{n}\sum\:_{j=1}^{n}{\alpha\:}_{i}{\alpha\:}_{j}{y}_{i}{y}_{j}K({x}_{i},{x}_{j})$$

Subject to:

31 $$\:0\le\:{\alpha\:}_{i}\ge\:C\:\:\:and\:\:\:\sum\:_{i=1}^{n}{\alpha\:}_{i}{y}_{i}=0$$

The decision function for a new sample $\:x$is given by:

32 $$\:f\left(x\right)=sign\left(\sum\:_{i=1}^{n}{\alpha\:}_{i}{y}_{i}K\left({x}_{i},\:x\right)+b\right)$$

In various studies, the use of machine learning algorithms for predicting the characteristics of oil and gas reservoirs, especially in the hydraulic fracturing process, has been explored. For instance, in 2022, Kamali et al.^70^ utilized machine learning models to predict permeability in carbonate reservoirs and simulated the GMDH model as the most accurate one. Additionally, in 2024 the study by Feng et al.,^71^ the CNN model demonstrated the best performance in predicting groundwater levels, which could similarly predict fluid behavior in hydraulic fracturing reservoirs. In 2021, Barjouei et al.^72^ applied deep learning algorithms to predict liquid flow rates through oil wells, showing that deep learning models outperformed other models in terms of accuracy. These studies highlight the potential of machine learning algorithms, particularly deep learning models, in predicting reservoir characteristics and optimizing the hydraulic fracturing process.

In their 2023 study, Ghorbani et al.^73^ used similar algorithms like RF and SVM for predicting coronary artery disease, identifying the RF model as the most accurate. The use of machine learning algorithms like RF for predicting complex reservoir features can improve the accuracy of predictions in processes related to oil and gas extraction, such as hydraulic fracturing. These studies demonstrate that advanced machine learning models, especially in complex environments like carbonate and oil reservoirs, can serve as valuable tools for predicting and optimizing production processes.

## Results and discussion

### Data division into training and testing sets

At the beginning of this study, all collected input parameters were carefully analyzed and reviewed. Subsequently, the input parameters, including $\:\mu\:$, $\:h$, $\:X$, and $\:t$, were selected for performing computations using ML algorithms in MATLAB software. The input data were then divided into two separate sets, namely training and testing datasets, to evaluate the performance of the algorithms. The proposed algorithms for HF were executed based on different ratios of training to testing data, and the accuracy of each method was calculated and presented in graphical form.

The random selection of input data based on these ratios can significantly affect the final accuracy of the algorithms. Accordingly, each method was evaluated through 10 independent runs, and the average R^2^ values obtained from these runs were reported as the final results. This approach provides a precise assessment of the performance and accuracy of each algorithm.

Table 3 reports the final R² values for each layer within the range of 0.1 to 0.9. These values are independently calculated for each ML method and are presented for the test, train, and test/train data sets. In fact, based on the results in the test/train rows, the optimal models can be selected.

**Table 3:** Final R2 values for each layer across ML Methods.

		*R*^2^-NN	*R*^2^-RF	*R*^2^-SVM
Test	10%	0.5624	0.5513	0.5450
	20%	0.8629	0.8526	0.8495
	30%	0.9630	0.9592	0.9573
	40%	0.9628	0.9630	0.9622
	50%	0.9632	0.9655	0.9653
	60%	0.9632	0.9675	0.9686
	70%	0.9631	0.9734	0.9735
	80%	0.9631	0.9770	0.9761
	90%	0.9634	0.9768	0.9779
Train	90%	0.5738	0.5890	0.5779
	80%	0.8723	0.8874	0.8761
	70%	0.9711	0.9849	0.9771
	60%	0.9687	0.9860	0.9773
	50%	0.9686	0.9842	0.9794
	40%	0.9676	0.9848	0.9796
	30%	0.9666	0.9850	0.9793
	20%	0.9662	0.9838	0.9801
	10%	0.9647	0.9817	0.9810
Test/Train	10–90%	0.5681	0.5698	0.5612
	20–80%	0.8676	0.8698	0.7627
	30–70%	0.9670	0.9720	0.9672
	40–60%	0.9657	0.9745	0.9697
	50–50%	0.9659	0.9748	0.9723
	60–40%	0.9654	0.9761	0.9741
	70–30%	0.9649	0.9792	0.9764
	80–20%	0.9647	0.9804	0.9781
	90–10%	0.9641	0.9792	0.9795

**Fig. 7:**
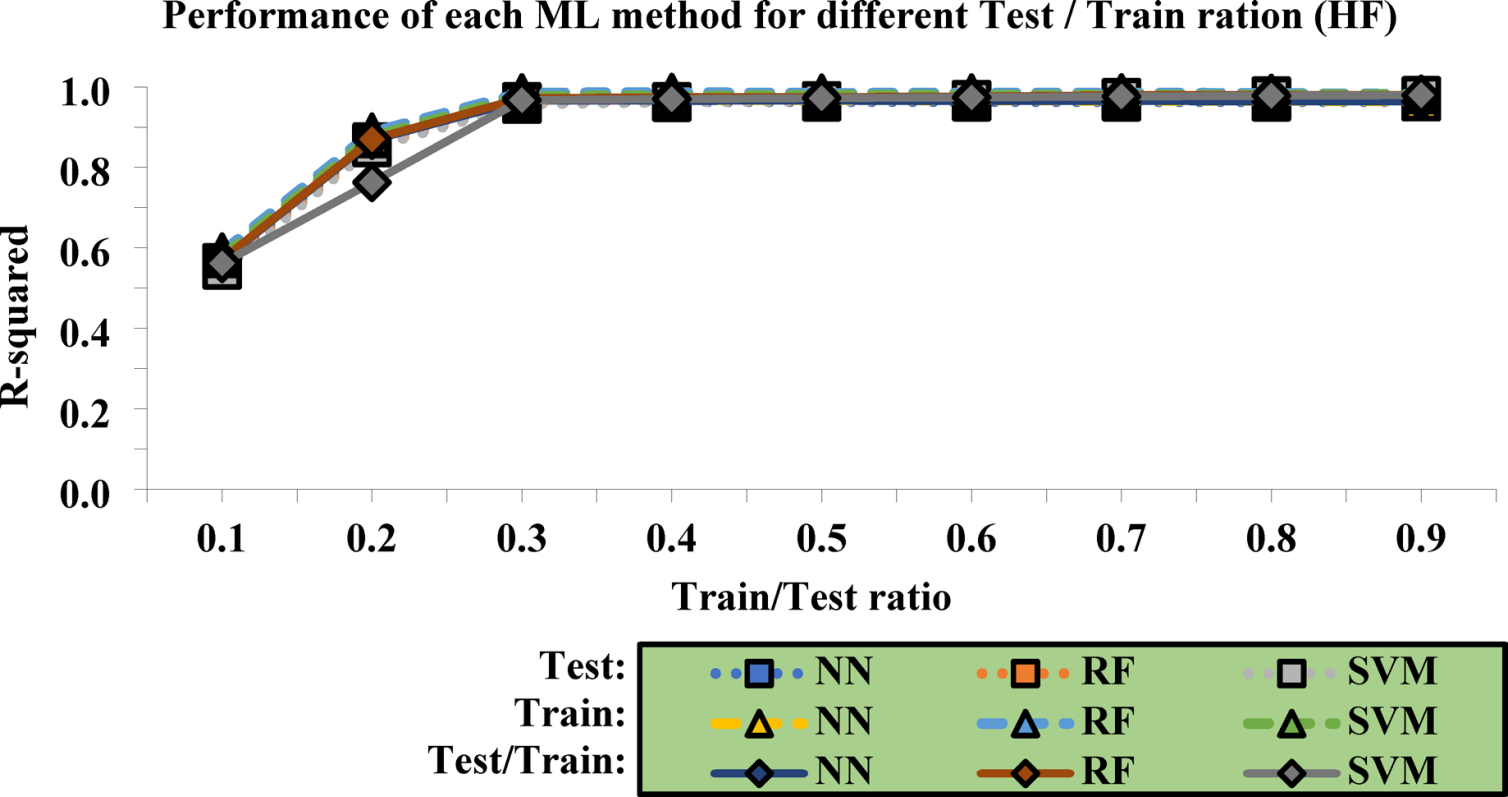
Performance of each ML method for different Train/Test ration (HF).

The optimal performance of each ML method is presented in graphical form in Fig. 7. which includes both training and testing data. This figure provides a clear visual representation of how each method performs under different conditions. By examining the graphical data, it becomes evident which methods are more reliable and effective in achieving accurate predictions, allowing for a more in-depth understanding of their performance across various datasets. The comparison between training and testing data also allows for the assessment of overfitting or underfitting, which is crucial for determining the generalizability of the models.

**Fig. 8:**
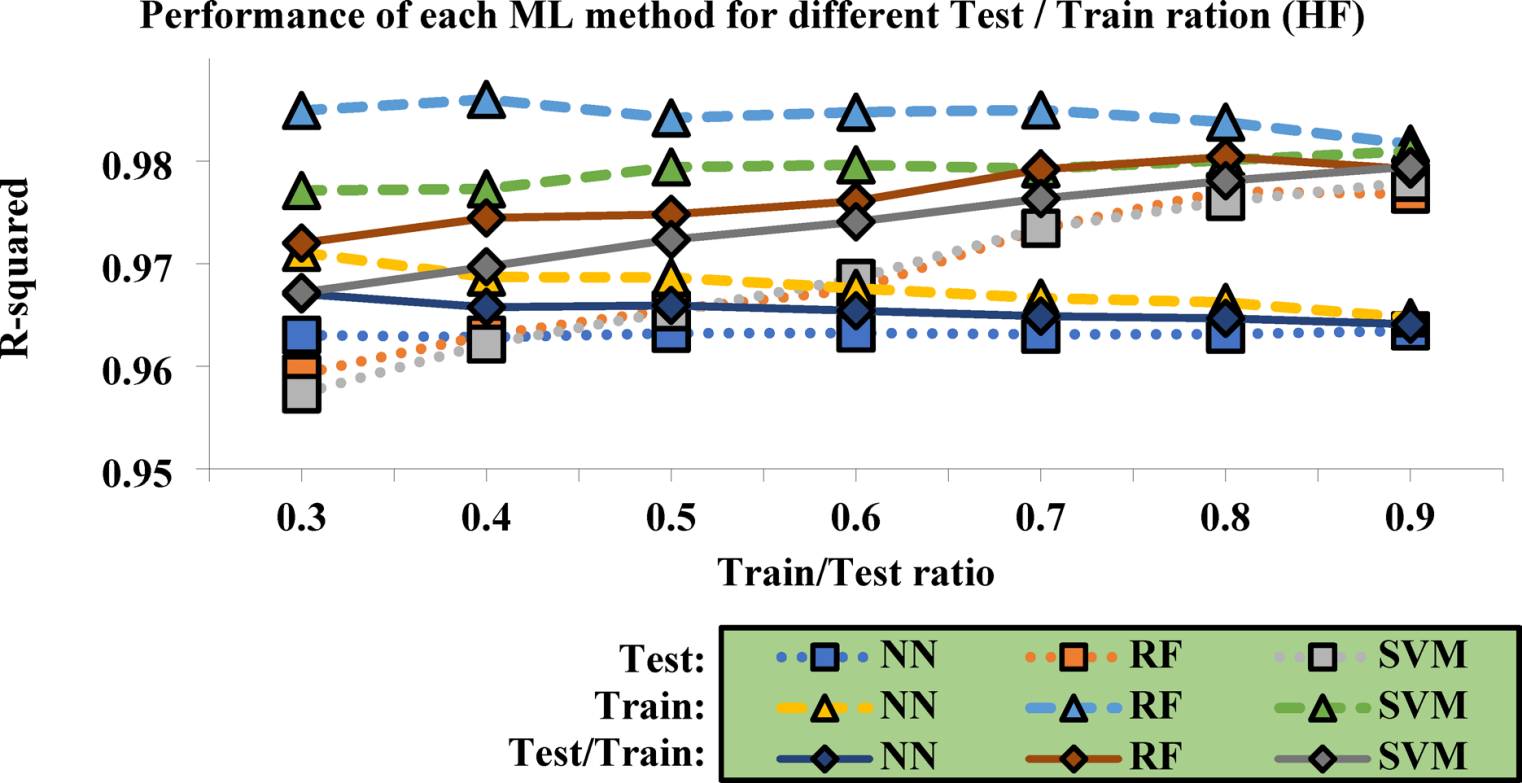
Performance of each ML method for different Train/Test ratios with limitations.

To conduct a more precise and comprehensive evaluation of the algorithms’ performance, the ranges considered in the figure were narrowed to enable more accurate and focused analyses (See Fig. 8). By refining the scope of the data, the results can be better interpreted, allowing for a deeper understanding of the algorithms’ behavior. Specifically, the Train/Test ratio was set within a range of 0.3 to 0.9, which provided a balance between training and testing data that is essential for assessing the models’ generalization capabilities. Additionally, the R-squared values were confined to the range of 0.95 to 0.99 to ensure that the analysis focused on the most accurate models, thus eliminating any results that might indicate poor model fit or excessive variance.

These adjustments were crucial in facilitating a more detailed and refined analysis of the performance metrics. By narrowing the focus to these specific ranges, it became easier to discern patterns and trends in the data that would have otherwise been obscured by broader ranges. This allowed for the identification of the optimal conditions under which each method performed best, making the evaluation process more insightful.

Based on the results presented in Fig. 8, the R^2^ values further corroborate the relationship between the algorithms’ accuracy and the conditions of the training-to-testing data ratio. The findings reveal that each algorithm has an ideal ratio that maximizes its predictive power. Specifically, for the NN, the highest accuracy was achieved with a training-to-testing ratio of 0.7, while the RF performed best at a ratio of 0.8, and the SVM reached its peak performance at a ratio of 0.9. These observations underscore the sensitivity of each algorithm’s performance to the ratio of training to testing data, emphasizing the importance of fine-tuning this parameter during the modeling process. By optimizing this aspect, it is possible to enhance the overall performance of the models, ensuring that they are not only accurate but also robust across different datasets.

Subsequently, the R^2^ values for each ML method will be examined in greater detail, providing a comprehensive analysis of their performance.

### Performance of each method in the training and testing phases

The results obtained from the regression analyses and R^2^ values are presented in graphical charts, where the data from the training (Train) and testing (Test) sets are shown simultaneously. These charts clearly display the model’s fit to the data, with the final R^2^ value accurately depicted. This method is particularly useful for evaluating the model’s accuracy and performance in predicting test data, and it also facilitates the comparison of the model’s performance across the training and testing datasets.

**Fig. 9:**
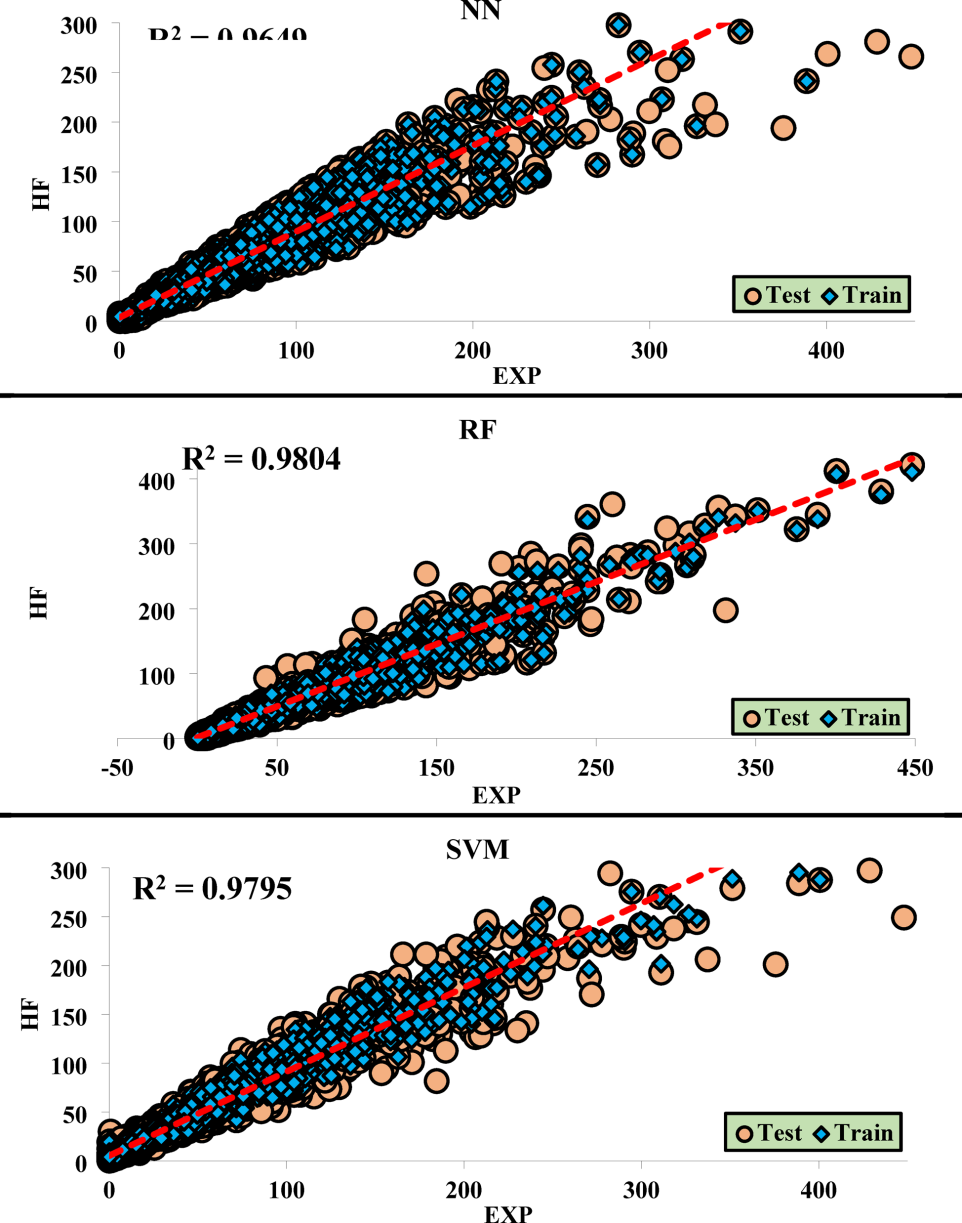
Regression of Different ML Methods for HF.

Figure 9 presents the obtained R^2^ values from HF.

Table 4 presents the regression equations for predicting the output based on a specific input variable. These equations have been extracted using various ML methods, with the goal of modeling and simulating the mathematical relationships between input and output variables. In this study, these equations have been specifically designed for analyzing and predicting the behavior of different systems. Through these methods, high-accuracy models have been developed to establish the relationship between inputs and outputs, which can be effectively used in decision-making and process optimization. To ensure transparency and reproducibility, the default control parameters used for each machine learning algorithm in MATLAB are presented in Table 5.

**Table 4:** The regression equations for each of the ML methods.

Equations	NN	Test	Output ~ = 0.84*Target + 4.6
		Train	Output ~ = 0.86*Target + 3.6
	RF	Test	Output ~ = 0.97*Target + 1.4
		Train	Output ~ = 0.97*Target + 1.3
	SVM	Test	Output ~ = 0.91*Target + 4.2
		Train	Output ~ = 0.9*Target + 4

**Table 5:** Control parameters of RF, SVM, and NN algorithms used in MATLAB.

Algorithm	Parameter	Default Value	Description
Random Forest (RF)	Method	Bag	Bagging method used to construct the ensemble
	NumLearningCycles	100	Number of decision trees
	Learners	Tree	Type of base learner
	MinLeafSize	1	Minimum number of observations per leaf
Support Vector Machine (SVM)	KernelFunction	linear	Type of kernel function
	BoxConstraint (C)	1	Penalty parameter of the error term
	KernelScale	auto	Automatically scaled kernel
	Standardize	false	No data standardization applied
Neural Network (NN)	HiddenLayerSize	10	Number of neurons in the hidden layer
	Training Function	trainlm	Levenberg–Marquardt backpropagation
	Performance Function	mse	Mean squared error loss function
	Epochs	1000	Maximum number of training iterations
	Goal	0	Training goal; 0 means continue until other stopping criteria are met

### Statistical criteria in measuring the accuracy of machine learning methods

In conclusion, the accuracy and performance of each method presented in this article have been compared using statistical metrics such as the correlation coefficient and the mean relative error. The following equations outline the calculation methods for these statistical parameters. Table 6 presents the performance values for each method.

The RMSE is a key statistical measure used to evaluate the effectiveness of predictive models. RMSE measures the difference between predicted values and actual values, providing an indication of the model’s accuracy. It is typically used to assess model performance by calculating the average squared error. The RMSE value ranges from zero to infinity, with values closer to zero indicating higher model accuracy.

33 $$\:RMSE=\sqrt{\frac{1}{n}\sum\:_{i=1}^{n}{\left({y}_{i}-{\widehat{y}}_{i}\right)}^{2}}$$

The Mean Absolute Deviation (MAD) is a measure used to assess the accuracy of prediction models by averaging the absolute differences between the actual and predicted values. A lower MAD value signifies higher model accuracy, and a MAD of zero indicates perfect alignment between the model’s predictions and the real data. This metric is especially valuable for comparing the performance of different models and optimizing them.

34 $$\:MAD=\frac{1}{n}\sum\:_{i=1}^{n}\left|{y}_{i}-{\widehat{y}}_{i}\right|$$

In this equation, $\:{y}_{i}$ denotes the actual value for sample $\:i$, while $\:{\widehat{y}}_{i}\:$represents the predicted value for the same sample. indicates the total number of samples. This formula considers both positive and negative errors as absolute values, meaning all errors are treated as positive, which helps minimize the effect of large errors.

R^2^ is a statistical measure used to evaluate the effectiveness of prediction models, often serving as a reliable criterion for model assessment. Also known as the coefficient of determination, R^2^ quantifies the proportion of variation in the dependent variable that can be explained by the independent variables in the model’s predicted results.

A high R^2^ value, close to 1, indicates that the model has successfully accounted for most of the variation in the dependent variable, resulting in highly accurate predictions. On the other hand, a low R^2^ value, approaching 0, suggests that the model has not effectively captured the variations in the dependent variable, leading to significant discrepancies between the predicted and actual values.

35 $$\:{R}^{2}=1-\frac{\sum\:_{i=1}^{N}{({y}_{i}^{Pred}-{y}_{i}^{exp})}^{2}}{\sum\:_{i=1}^{N}{({y}_{i}^{Pred}-{average(y}_{i}^{exp}))}^{2}}$$

**Table 6:** Plotted error values of ML methods.

	NN		RF		SVM	
	Test	Train	Test	Train	Test	Train
MAD	7.162	6.933	6.366	5.878	6.588	5.384
RMSE	14.706	13.437	11.929	10.48	13.162	10.581

**Fig. 10:**
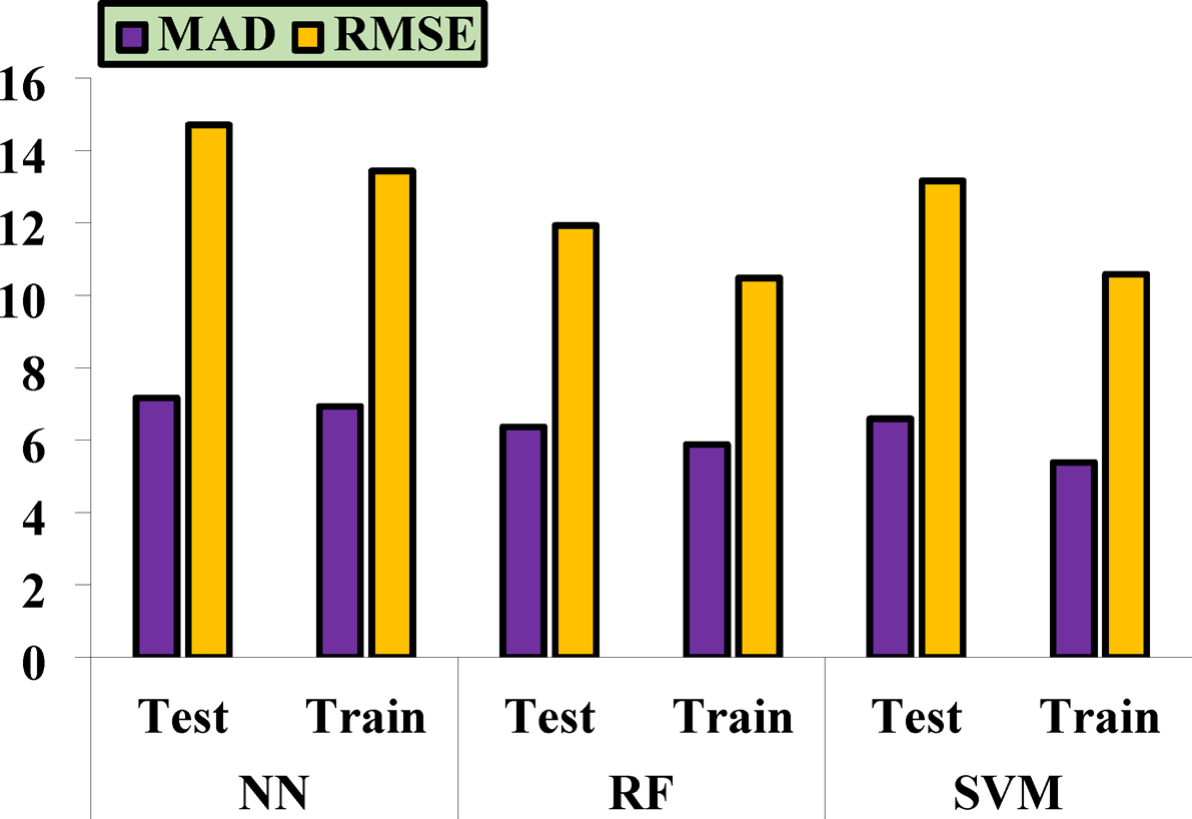
The mean absolute error of simulation, correlation, and ML of HF for the validation data.

Table 6 presents the error values for each ML method, providing a precise numerical comparison of the performance of each model. This table offers key information that allows for a quantitative evaluation of the accuracy of the predictions made by each method. In contrast, Fig. 10 graphically displays the errors for RMSE and MAD, which are particularly useful for directly comparing the performance of the methods. This graphical representation clearly illustrates the differences in the predictive accuracy of each model, helping to better understand the deviations between predicted and actual values. By using these two-error metrics, RMSE and MAD, it becomes easier to evaluate the performance of each method against the test data, allowing for a clearer comparison.

Table 7 presents the results of the best method (RF) for predicting the HF characteristics. This table reports various metrics for evaluating the model’s accuracy, including the following:

**Table 7:** Best results for each of the HF.

Best Method	*R* ^2^	MAD (Test)	MAD (Train)	RMSE (Test)	RMSE (Train)
RF	0.9804	6.366	5.878	11.929	10.48

- **R**^**2**^: The coefficient of determination, which indicates the degree of fit between the model and the actual data. For the RF method, it is 0.9804, reflecting the high accuracy of the model.
- **MAD**: The mean absolute deviation, reported as 6.366 for the test data and 5.878 for the training data.
- **RMSE**: The root mean squared error, which is 11.929 for the test data and 10.48 for the training data.

These values indicate the excellent performance of the RF method in prediction, particularly given the high R^2^ value and relatively low errors for MAD, and RMSE.

**Fig. 11:**
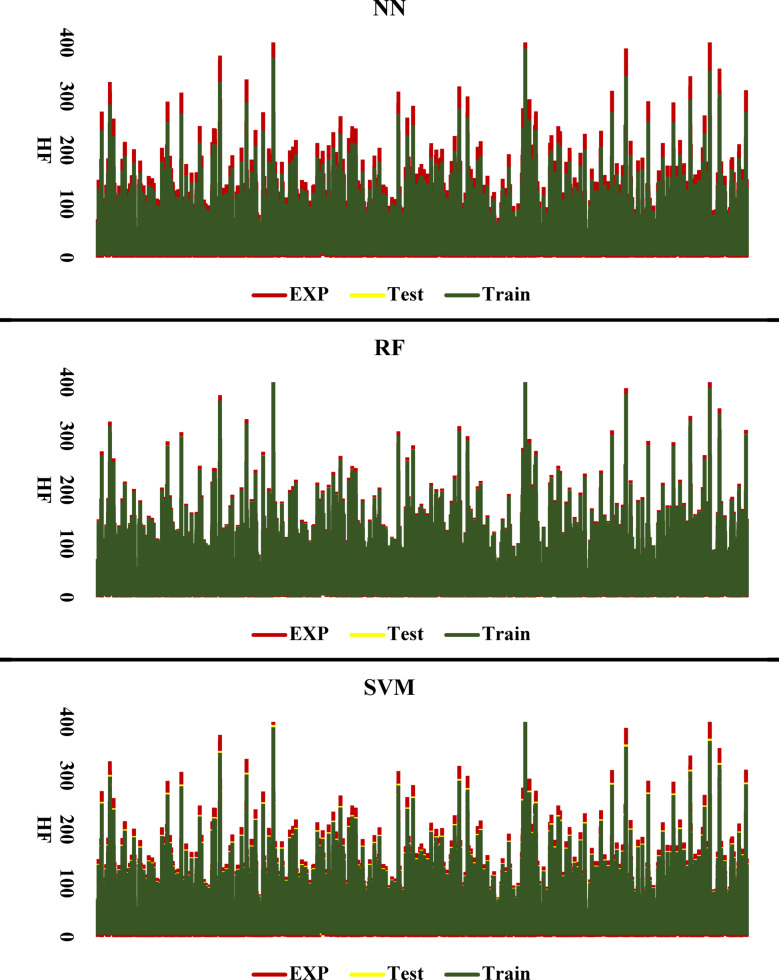
Comparison of algorithms with laboratory results.

To validate the performance of the machine learning algorithms, the results obtained from the hydraulic failure evaluation using these algorithms were sent to the laboratory method for comparison. The goal was to conduct a precise and comprehensive comparison to determine which of the algorithms best aligns with the experimental method. Specifically, the comparison focused on the level of agreement between each algorithm’s predictions and the real laboratory data. By employing various evaluation metrics and carefully examining the differences between predicted values and actual observations, the results from each algorithm were continuously compared with the laboratory data. Ultimately, it was observed that the RF method demonstrated the best performance in aligning with the laboratory data, providing predictions that closely matched the real results. This finding confirms that, as mentioned throughout the article, RF is one of the most effective algorithms in this context and exhibits the best response compared to the other methods. These results highlight the importance of selecting the right algorithm for modeling and prediction, especially when high accuracy and close alignment with experimental data are required (See Fig. 11).

Table 8 Summarizes the main limitations identified in the current study. Although the developed machine learning models demonstrated high predictive accuracy for estimating HF depth, several challenges remain. These include the dependency on field-specific data, limited generalization across other geological settings, and the absence of uncertainty quantification in the predictive outputs. Additionally, the model’s performance May be influenced by the quality and completeness of the input features, and the lack of real-time validation restricts its immediate field applicability. Acknowledging these limitations provides transparency in model evaluation and highlights important directions for future research and improvement.

**Table 8:** Limitations of the present Study.

Limitation	Description
Data dependency	The model is trained and validated on a specific dataset, which may not represent all geological conditions or reservoir types.
Limited generalization	The model may not perform well when applied to other regions or datasets not included in the training process.
Small or imbalanced dataset	If the dataset is small or imbalanced (e.g., few high-depth vs. many low-depth cases), the model may produce biased results.
Feature selection bias	Some important geological or operational variables might be missing, leading to incomplete modeling of the HF process.
Model interpretability	Some ML models (e.g., ensemble or deep learning models) are difficult to interpret, which limits their acceptance by domain experts.
Overfitting	The model may perform well on the training data but poorly on unseen data, especially if regularization or validation was insufficient.
Lack of uncertainty quantification	Predictions are deterministic and do not include confidence intervals or uncertainty estimates.
Assumption of data quality	The model assumes that input data is accurate and free from noise, which may not reflect field reality.
No real-time validation	The model was not validated in real-time or in real field applications, so its practical performance is unknown.
Computational cost	Some models may require significant computational resources for training and tuning.
Lack of sensitivity analysis	The influence of input parameters on the output is not fully explored or quantified.
Temporal static assumption	The model assumes static relationships, while subsurface properties or operational parameters may change over time.

## Conclusions

This study presented a comprehensive and data-driven machine learning (ML) framework for evaluating and predicting the performance of hydraulic fracturing (HF) operations. By integrating three widely used ML algorithms—Random Forest (RF), Support Vector Machine (SVM), and Neural Networks (NN)—and applying them to a large-scale dataset comprising 16,000 records, the study successfully addressed the limitations of traditional physics-based models in capturing the complex and nonlinear relationships among fracturing parameters.

Among the tested models, the RF algorithm demonstrated the best overall performance with a coefficient of determination (R^2^) of 0.9804, a Mean Absolute Deviation (MAD) of 6.366 (test) and 5.878 (train), and a Root Mean Square Error (RMSE) of 11.929 (test) and 10.48 (train). These metrics underscore the robustness and predictive accuracy of the RF model in modeling subsurface behavior and fracture geometry.

1. Large-Scale Dataset Usage: The use of a 16,000-point dataset, significantly larger than those used in previous research, improved model training and allowed for better generalization to unseen data.
2. Advanced Statistical Characterization: The input parameters were statistically analyzed using box plots, violin plots, and descriptive statistics (mean, variance, skewness, kurtosis, quartiles). This detailed analysis supported the understanding of variable distributions and ensured improved model reliability.
3. Systematic Evaluation Across Train/Test Splits: The study uniquely examined model performance over varying train/test ratios (from 0.1 to 0.9), revealing how data availability influences accuracy and enabling the identification of optimal training conditions for each algorithm.
4. Physics-Informed Feature Selection: Unlike purely data-driven models, the selected features were grounded in physical principles of HF—such as fracture height, injection time, crack length, and fluid viscosity—linking machine learning models to engineering relevance.
5. Comparative Model Evaluation: All three ML algorithms were evaluated under identical conditions to ensure fairness in comparison. RF consistently outperformed NN and SVM, particularly in lower error rates and higher R² values, highlighting its suitability for HF prediction tasks.

The outcomes of this study present a scalable and transferable ML-based framework that can support field engineers and decision-makers in optimizing HF designs. By reducing prediction error and computational costs, the proposed models—especially RF—can be integrated into real-time systems for hydraulic fracturing optimization in heterogeneous reservoirs. However, the study also acknowledges several limitations, such as the lack of real-time validation, dependency on dataset quality, and generalization constraints across different geological settings.

Future research may focus on expanding the dataset to include additional geological and operational parameters, incorporating uncertainty quantification (e.g., confidence intervals), and deploying the models in real-time field scenarios to validate their practical performance.

In conclusion, this work not only highlights the power of machine learning in hydraulic fracturing prediction but also demonstrates how intelligent modeling, when combined with domain knowledge and statistical rigor, can pave the way for data-driven reservoir engineering and more efficient hydrocarbon recovery strategies.

## Data Availability

The datasets used and/or analyzed during the current study available from the corresponding author on reasonable request.

## Greek letters

$\mu$: Viscosity of the fracturing fluid (cP):
${\xi }_{i}$: Slack variable in optimization:
$${\sigma }^{2}$$: Population variance
$\gamma$: Parameter in RBF kernel

## Latin letters

h: Crack height
t: Injection time (s)
X: Crack length (ft)
Q1: First quartile
Q3: Third quartile
$${s}^{2}$$: Sample variance

## Abbreviations

ANN: Artificial Neural Network
B-DNN: Branched Deep Neural Network
BPNN: Back Propagation Neural Network
CNN: Convolutional Neural Network
GRU: Gated Recurrent Unit
HF: Hydraulic Fracturing
MAD: Mean Absolute Deviation
MAPE: Mean Absolute Percentage Error
ML: Machine Learning
MLR: Multiple Linear Regression
NN: Neural Networks
PSO: Particle Swarm Optimization
RBF: Radial Basis Function
RMSE: Root Mean Square Error
RF: Random Forest
RNN: Recurrent Neural Network:
SVM: Support Vector Machine
